# Exposure/Risk Assessment of Employees in Gasoline Refueling Stations with and Without the Efficacy of Vapor Recovery Systems in Mexico

**DOI:** 10.3390/ijerph22010010

**Published:** 2024-12-25

**Authors:** Naohide Shinohara, Jose Juan Felipe Ángeles García, Miguel Magaña Reyes, Becki Gatica Jiménez, Roberto Basaldud Cruz, Beatriz Cardenas Gonzalez, Shinji Wakamatsu

**Affiliations:** 1 Research Institute of Science for Safety and Sustainability (RISS), National Institute of Advanced Industrial Science and Technology (AIST), 16-1 Onogawa, Tsukuba, Ibaraki 305-8569, Japan; 2 National Institute of Ecology and Climate Change (INECC), The Secretariat of Environment and Natural Resources (SEMARNAT), Blvd. Adolfo Ruíz Cortines 4209 Jardines en la Montaña, Ciudad de México 14210, Mexico; jjfangeles@yahoo.com.mx (J.J.F.Á.G.); mmagana@ine.gob.mx (M.M.R.); j_g_becki@yahoo.com.mx (B.G.J.); roberto.basaldud@inecc.gob.mx (R.B.C.); 3 World Resources Institute Mexico, Belisario Dominguez No. 8, Coyoacan, Ciudad de México 04000, Mexico; beatriz.cardenas@wri.org; 4 Institute of Integrated Atmospheric Environment, 1-2-8 Koraku, Bunkyo, Tokyo 112-0004, Japan; wakamatu@agr.ehime-u.ac.jp; 5 Faculty of Agriculture, Ehime University, 10-13 Dogo-himata, Matsuyama, Ibaraki 790-8577, Japan

**Keywords:** gas station, personal exposure, vapor recovery system, BTEX, formaldehyde, carbon monoxide

## Abstract

Concerns regarding the health risks associated with employe exposure to volatile chemicals during gasoline refueling necessitates rigorous investigation and effective countermeasures. This study aims to evaluate the efficacy of vapor recovery systems in mitigating exposure risks during gasoline refueling. Employee exposure to volatile organic compounds, aldehydes, carbon monoxide, and fine particulate matter (PM_2.5_) was assessed at gasoline stations with and without vapor recovery systems. Three stations each from the State of Mexico and Mexico City, equipped with gasoline vapor recovery systems, were compared with three stations in Guadalajara lacking such systems. The exposure concentrations (mean ± standard deviation) to benzene in Guadalajara, the State of Mexico, and Mexico City were 45 ± 29, 24 ± 20, and 18 ± 15 μg/m^3^, respectively, which were significantly higher than the background atmospheric concentrations at 1.6 ± 0.56, 0.72 ± 0.083, and 0.65 ± 0.14 μg/m^3^, respectively. Similarly, the exposure concentrations of toluene, ethylbenzene, and xylenes at gasoline stations were significantly higher than the background atmospheric concentrations. However, the exposure concentrations of formaldehyde and PM_2.5_ were similar to the background atmospheric concentrations. The excess cancer risks due to benzene exposure were estimated at 1.2–4.2 × 10^−5^, 0.63–2.2 × 10^−5^, and 0.46–1.6 × 10^−5^ (mean) and 0.42–1.5 × 10^−4^, 0.29–1.0 × 10^−4^, and 2.4–8.6 × 10^−5^ (maximum) in Guadalajara, the State of Mexico, and Mexico City, respectively. The risk to employees in gasoline stations was reduced by 47–61% in service stations with gasoline vapor recovery systems.

## 1. Introduction

Volatile organic compounds (VOCs) present in gasoline evaporate from the gasoline tanks of vehicles while refueling. Gasoline evaporation can cause economic losses and have adverse effects on employee health. Benzene, toluene, ethylbenzene, and xylene (BTEX) are the major VOCs that evaporate from gasoline. Benzene is a carcinogen [1]. Toluene, ethylbenzene, and xylene induce similar toxicities, including neurological effects [2,3,4,5,6,7]. Cars release formaldehyde, carbon monoxide (CO), and fine particulate matter (PM_2.5_). Formaldehyde is a carcinogen [1] that induces eye and nose irritation, as well as respiratory effects [8,9,10]. CO has also been reported to have reproductive effects [11,12]. PM_2.5_ is associated with respiratory, circulatory, and lung cancers [13]. Exposure to gasoline or gasoline components may cause various adverse health effects including cancer [14], premature deaths, and years of life lost [15], and some studies have suggested that safety measures are needed [16].

VOCs are emitted during gasoline station refueling, and the risk of exposure is a concern [17,18]. The health risk from gasoline station customer exposure is considered negligible [18,19], whereas the risk to employees needs to be addressed [20]. Exposure to benzene at gasoline stations has been reported to be 163,723 μg/m^3^ in the Middle East, South America, and Southeast Asia [21,22,23], while exposure concentrations much lower than these have been reported in the United States and Japan (10.4–57.3 μg/m^3^) [18,24]. It is not known what the exposure concentrations are at gasoline stations in Mexico.

Studies have also suggested that the risk of inhalation of submicron particles by employees at gasoline stations is high [25]. Sultana and Hoover identified BTEX, propylene, hexane, and formaldehyde as gasoline-derived substances with high emission and toxicity concerns in California [26]. While CO exposure, a commonly acknowledged hazardous substance emitted from motor vehicles, has received considerable attention [27], vehicle emissions of CO have shown improvement recently [28]. However, the extent of CO exposure remains inadequately understood. VOC and CO emissions vary widely according to vehicle type [29] and gasoline composition [30], and their exposure may vary widely across countries and regions.

In the Mexico City metropolitan area, including the State of Mexico and Mexico City, 4.5 million vehicles were registered in 2008 [31]. To reduce atmospheric air pollution, the governments of Mexico City and the State of Mexico, in coordination with the federal government, developed and implemented the ProAire 2002–2010 [31,32] and ProAire 2011–2020 [33] programs, which mandated several action items under different themes. Therefore, significant reductions in commuter exposure to VOCs during transit in Mexico City have been reported [32,34]. In the Guadalajara metropolitan area, the second largest city in Mexico, approximately 3.0 million vehicles are used [33].

Countermeasures to reduce exposure to gasoline vapor during refueling include onboard refueling vapor recovery control (ORVR) attached to the vehicle side, which is mandatory in the USA [35,36], and the vapor recovery devices on the refueling nozzle side of gasoline stations (Stage II). In Japan, subsidies have been introduced for vapor recovery devices (Stage II) on the nozzle side of gasoline stations, and their installation has been promoted, but they have not yet been widely installed. Gasoline vapor recovery systems (Stage II) must be installed at gasoline stations in the State of Mexico and Mexico City [37]. In contrast, in the Guadalajara metropolitan area, gasoline vapor recovery systems remain absent in gasoline stations. Despite reports indicating that vapor recovery can reduce VOC emissions to atmosphere by 45% [38], the disparity in VOC exposure between gasoline stations with and without such systems remains unexplored.

In this study, we examined the personal exposure concentrations of VOCs, aldehydes, CO, and PM_2.5_ among employees at gasoline stations of varying sizes. Specifically, we compared stations in Guadalajara without vapor recovery systems with those in the State of Mexico and Mexico City, where vapor recovery systems were installed.

## 2. Materials and Methods

### 2.1. Survey Area and Period

Occupational exposure levels to VOCs, aldehydes, CO, and PM_2.5_ were assessed at three gasoline stations in Guadalajara (G1, G2, and G3) without vapor recovery systems, during May 2012 from 7:00 to 15:00. Similarly, measurements were taken at three gasoline stations in the State of Mexico (SM1, SM2, and SM3), equipped with vapor recovery systems, and three gasoline stations in Mexico City (CDMX1, CDMX2, and CDMX3), also with vapor recovery systems, during July 2015 from 9:00 to 17:00. These vapor recovery systems installed at gasoline stations in the State of Mexico and Mexico City were certified to comply with the vapor recovery standards established in Mexico, NOM-004-ASEA-2010. Background levels were determined at locations near the gasoline stations (GB: 250 m from G1 and 1 km from G2 and G3; SMB1, SMB2, SMB3, CDMXB1, CDMXB2, and CDMXB3: within 500 m of SM1, SM2, SM3, CDMX1, CDMX2, and CDMX3, respectively). Annual gasoline sales at the gas stations, data for which were provided by each gas station for this project, differed significantly in the three cities, in the following order G1 > G2 > G3; SM1 > SM2 > SM3; CDMX1 > CDMX2 > CDMX3. Sampling and measurements were conducted every 2 d over a period of 6 d on weekdays at each station. The number of cars refueled and the amount of money spent on fuel during the survey period were also provided by each gasoline station.

### 2.2. Sampling and Analysis

Employees were asked to wear pumps at all times, and sampling was carried out with cartridges, tubes, and filters attached to the collar, which is the breathing zone of the employee. For background measurements, cartridges, tubes, and filters were set at a height of 150 cm for collection. VOCs were sampled using a TENAX tube (Perkin Elmer Inc., Shelton, CT, USA) at a flow rate of 50 mL/min using a portable pump (pocket pump, SKC Inc., Eighty Four, PA, USA) for personal exposure and ambient samples. The aldehydes were sampled using a glass tube with ozone scrubbers and 2,4-dinitrophenylhydrazine (226–120, SKC Inc., USA) at 250 mL/min using a portable pump (AirChek XR5000 Sample Pump; SKC Inc., USA). The tubes were changed every 2 h to detect changes over time. The CO concentration was measured every 1 min using an electrochemical CO sensor (T15; Langan Products Inc., San Francisco, CA, USA). PM_2.5_ was sampled using a 37 mm Teflon filter in a single-stage impactor for 8 h (Model 200 PEM-4–2.5; MSP Corporation, Shoreview, MN, USA) at 4 L/min using a pump (224-PCXR8; SKC Inc., USA). The flow rates of the pumps were measured 10 times before and after sampling to test their stability. The average flow rates of 20 measurements were used to calculate the concentrations.

The VOC samples were thermally desorbed using a thermal desorption system (ATD400, PerkinElmer Inc., Shelton, CT, USA) and analyzed using a gas chromatography–flame ionization detector (PerkinElmer) in accordance with the United States Environmental Protection Agency (US EPA) method TO-17 [39]. The aldehyde samples were extracted using 5 mL of acetonitrile and analyzed using high-performance liquid chromatography (HP1100; Agilent Technologies, Inc., Santa Clara, CA, USA) according to the US EPA method 8315A [40]. The PM_2.5_ concentrations were analyzed based on the weight difference before and after sampling. The filter was weighed using an ultra-microbalance (CAHN C-35; Thermo Fisher Scientific, Waltham, MA, USA). To prepare the calibration curves of aldehydes and VOCs, a mixed standard solution of DNPH derivatives (TO11/IP-6A aldehyde/ketone-DNPH mix; CRM47285, Supelco) and a standard gas mixture (1 ppm BTEX standard-2 [Linde Gas North America LLC, Danbury, CT, USA] or PAMS-J58 [Sumitomo Seika Chemicals Co., Ltd., Osaka, Japan]) were used.

The calibration curves for formaldehyde and VOCs were linear in the range between 0.018 and 0.55 μg/mL (R^2^ > 0.998) and between 0.007–0.02 and 1.7–4.5 μg/tube (R^2^ > 0.995), respectively. The limit of quantification was determined as the greater of 10 times the standard deviation of 6 travel blanks or the lower limit of the calibration curve linearity. The limit of quantification for formaldehyde was 2.4–4.5 μg/m^3^. The detection and quantification limits for benzene, toluene, ethylbenzene, m/p-xylene, and o-xylene were 5.9–7.9, 13–18, 4.8–6.4, 11–15, 7.7–10 μg/m^3^, respectively. For PM_2.5_, the quantification limits were 16–19 μg/m^3^. The analytical precision for formaldehyde at 0.1 ppbv was 0.65–3.6%. For benzene, toluene, ethylbenzene, m/p-xylene, and o-xylene, the accuracy was less than 15% at all times.

### 2.3. Risk Assessment of Employees

The excess cancer risks among gasoline station employees were assessed according to the US EPA guideline [41] using the following equation:

(1) $$excessRISK=UR\times C_{exp}\times \frac{H_{exp}\times D_{exp}\times Y_{exp}}{24\times 7\times 70}$$

where *excessRISK* is the excess lifetime cancer risk [unitless], *UR* is the lifetime carcinogenic unit risk [/(μg/m^3^)], *C_exp_* is the exposure concentration [μg/m^3^], *H_exp_* is the exposure hours per day [h/d], *D_exp_* is the exposure days per week [d/week], and *Y_exp_* is the exposure years per life [year]. A lifetime carcinogenic unit risk of 2.2 × 10^−6^ to 7.8 × 10^−6^/(µg/m^3^) for benzene inhalation as recommended by the US EPA was employed for the risk calculation [42]. Taking into account the working hours of the gasoline station employees, exposure durations of 8 h/d, 5 d/w, and 35 years were assumed for *H_exp_*, *D_exp_* and *Y_exp_*. Excess cancer risks were not evaluated for formaldehyde because employee exposure concentrations were not clearly different from those of the background levels.

Assuming that the mean and maximum exposure concentrations (2 h) obtained in this study would be the same as the average exposure concentrations in an 8 h working day, the non-carcinogenic risk was estimated by comparing the exposure concentrations with the TLV-TWA (8 h), which is the concentration at which no adverse effects occur throughout the occupational lifetime, assuming an average working day of 8 h. The hazard quotient values for toluene, ethylbenzene, xylenes, and CO were calculated using the following equation:

(2) $$HQ=\frac{C_{exp}}{TLV-TWA\;(8h)}$$

where *HQ* is the hazard quotient [unitless], *C_exp_* is the exposure concentration [μg/m^3^], and *TLV-TWA* (8 h) is the threshold limit value–time-weighted average for 8 h [μg/m^3^]. The *TLV-TWA* (8 h) set by the American Conference of Governmental Industrial Hygienists (ACGIH) of 75,000, 435,000, 435,000 μg/m^3^, and 25 ppm for toluene, ethylbenzene, xylenes, and CO, respectively, were used in this study. Xylene was evaluated using the total concentration of the m/p and o isomers.

There is a possibility of accidental dermal exposure, such as gasoline splashing on the body when refueling, but this study only considered inhalation exposure. The possibility of dermal exposure to gasoline vapors was treated as negligible, given that the workers wore long-sleeved work clothes.

## 3. Results

### 3.1. Refueling Volume and Number of Cars

**Figure 1:**
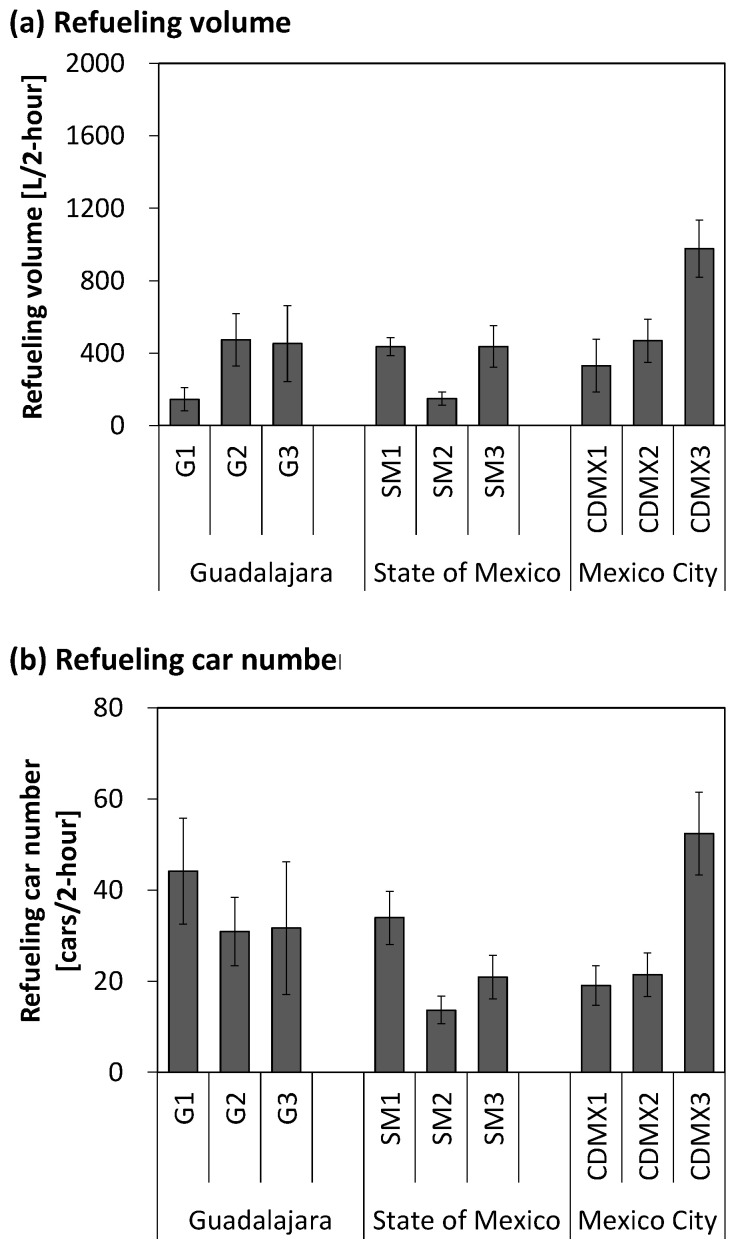
(**a**) Refueling volume of gasoline and (**b**) number of refueled cars for 2 h per employee. G: Guadalajara; SM: State of Mexico; CDMX: Mexico City (Ciudad de México).

The volume of gasoline refueled and number of cars refueled by an employee whose exposure levels were surveyed are shown in Figure 1, Figures S1 and S2. These values do not correlate with the gasoline sales for the entire gasoline station. In Guadalajara, the State of Mexico, and Mexico City, the employee refueled 360 ± 210, 340 ± 160, and 590 ± 310 L of gasoline (mean ± standard deviation [SD]) in 2 h and 36 ± 13, 23 ± 9.6, and 31 ± 17 cars in 2 h, respectively.

### 3.2. BTEX and Aldehydes

**Figure 2:**
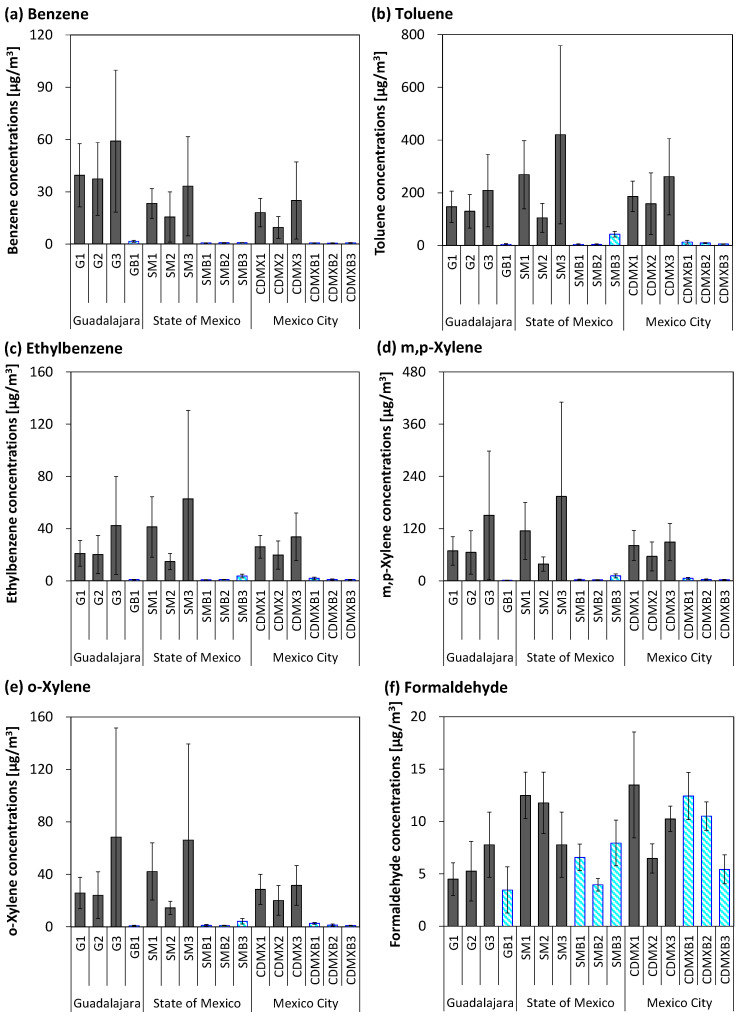
Personal exposure and background concentrations of volatile organic compounds at gasoline stations. (**a**) Benzene, (**b**) toluene, (**c**) ethylbenzene, (**d**) m,p-xylene, (**e**) o-xylene, and (**f**) formaldehyde. GB, SMB, and CDMXB indicate the background sampling locations in Guadalajara, the State of Mexico, and Mexico City. The bold bar indicates the mean value, and the thin line indicates the standard deviation (SD). Glay and blue bars indicate the personal exposure concentrations of employees and the background (outdoor) concentrations, respectively.

Personal exposure concentrations of employees to BTEX and formaldehyde are shown in Figure 2 and Figures S3–S5. Exposure levels did not differ between gasoline stations because the daily variations and differences among employees were high. No distinctive trends were observed according to sampling day or time of day. The exposure concentrations of benzene at gasoline stations (mean ± SD: Guadalajara: 45 ± 29 μg/m^3^, State of Mexico: 24 ± 20 μg/m^3^, and Mexico City: 18 ± 15 μg/m^3^) were significantly higher than the corresponding background atmospheric concentrations (Guadalajara: 1.6 ± 0.56 μg/m^3^, State of Mexico: 0.72 ± 0.083 μg/m^3^, and Mexico City 0.65 ± 0.14 μg/m^3^). Similarly, the exposure concentrations of toluene, ethylbenzene, and xylenes at gasoline stations were significantly higher than their background atmospheric concentrations. In contrast, exposure concentrations of formaldehyde (Guadalajara: 5.8 ± 2.9 μg/m^3^, State of Mexico: 11 ± 3.4 μg/m^3^, and Mexico City: 10 ± 4.2 μg/m^3^) did not differ from the corresponding background atmospheric concentrations (Guadalajara: 3.5 ± 2.2 μg/m^3^, State of Mexico: 6.2 ± 2.2 μg/m^3^, and Mexico City: 9.5 ± 3.5 μg/m^3^).

### 3.3. CO

**Figure 3:**
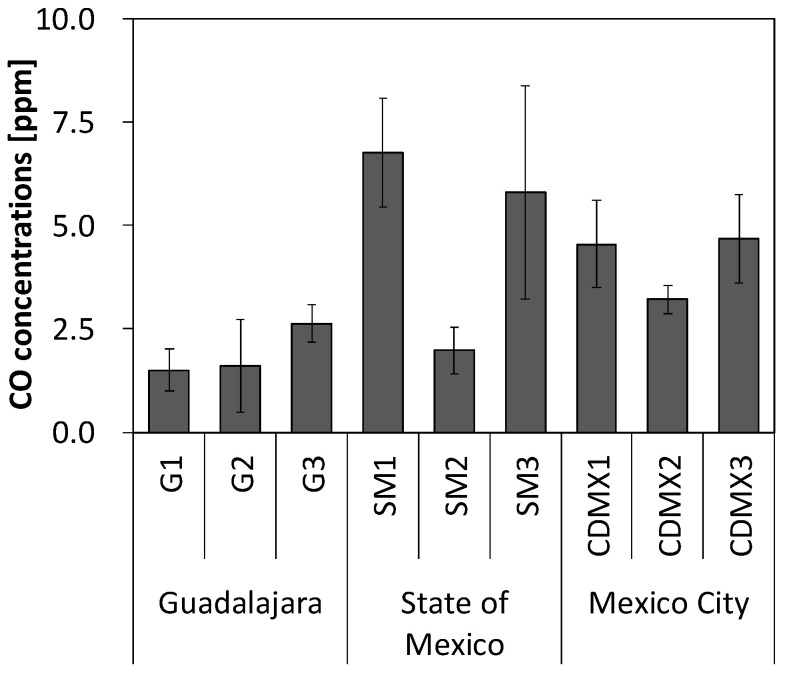
Personal exposure and background concentrations of carbon monoxide (CO) at gasoline stations. The bold bar indicates the mean value, and the thin line indicates the standard deviation (SD).

The personal exposure concentrations of the employees to CO are shown in Figure 3 and Figure S6. Minor differences were observed between the gasoline stations. Although there was no trend according to the day and time, the 1 min average concentration varied enormously, and there were many peaks of 2–5 min (Figure S7). Personal exposure concentrations to CO (mean ± SD) were 2.3 ± 1.2, 4.8 ± 2.6, and 4.1 ± 1.1 ppm at gasoline stations in Guadalajara, the State of Mexico, and Mexico City, respectively.

### 3.4. PM_2.5_

**Figure 4:**
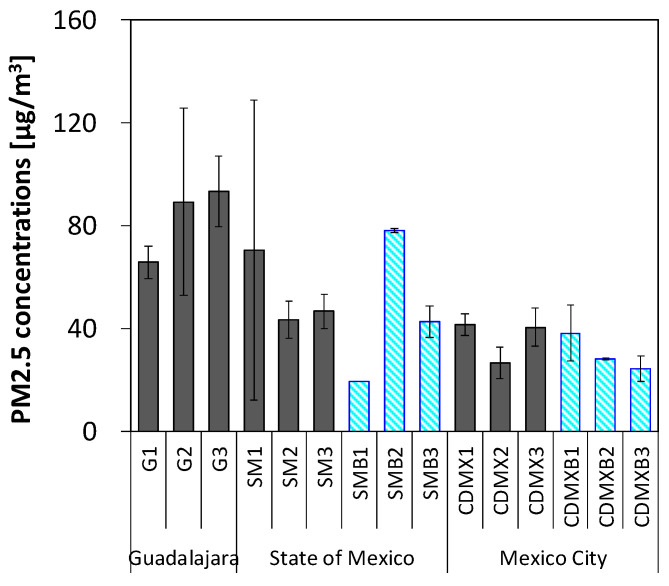
Personal exposure and background concentrations of PM_2.5_ at gasoline stations. The bold bar indicates the mean value, and the thin line indicates the standard deviation (SD). Glay and blue bars indicate the personal exposure concentrations of employees and the background (outdoor) concentrations, respectively.

The personal exposure concentrations of employees to PM_2.5_ are shown in Figure 4. Personal exposure to PM_2.5_ (83 ± 23, 51 ± 26, and 36 ± 8.9 μg/m^3^ at gasoline stations in Guadalajara, the State of Mexico, and Mexico City, respectively) did not differ from the corresponding background atmospheric concentrations (Guadalajara: no sample, State of Mexico: 52 ± 26 μg/m^3^, Mexico City: 30 ± 8.3 μg/m^3^).

### 3.5. Risk Assessment of Employees

The excess cancer risks of employees were estimated to be below 10^−4^ in all three cities when estimated using the average exposure concentrations, but it was 1.5 × 10^−4^ in Guadalajara when estimated from the maximum exposure concentration (Table 1). For non-carcinogenic compounds, even when using the maximum exposure concentrations, the HQ values for toluene, ethylbenzene, xylenes, and CO were 1.8 × 10^−2^, 3.0 × 10^−4^, 1.7 × 10^−3^, and 0.27, respectively.

## 4. Discussion

The exposure concentrations of BTEX were found to be significantly high compared to background atmospheric concentrations. BTEX, comprising gasoline vapor and exhaust gases from cars, including combustion and incomplete combustion gases, were predominant, whereas formaldehyde, CO, and PM_2.5_ primarily originated from vehicular exhaust gas. Since exposure concentrations to formaldehyde and PM_2.5_ showed no discernible difference from background concentrations, gasoline refueling was considered to have a minimal effect on the employees’ exposure to PM_2.5_ and HCHO. On the other hand, employee exposure to BTEX in gasoline stations was considered to be mostly due to the vapor of BTEX during gasoline refueling activities rather than vehicular exhaust gas. This conclusion aligns with findings from a previous study in Japan, where the real-time monitoring of total VOCs revealed a substantial increase in VOC exposure during refueling at gasoline stations [18]. Multiple peaks were observed where the exposure concentrations increased sharply for a few minutes, which may have been caused by exposure to exhaust gases emitted from vehicles stopping to refuel at gasoline stations.

The exposure concentrations of benzene at the gasoline stations in the State of Mexico and Mexico City, where gasoline vapor recovery systems were installed, were 0.53- and 0.39-times lower (21 and 28 μg/m^3^ lower) than those in Guadalajara, where gasoline vapor recovery systems were not installed. Due to higher background concentrations, greater refueling volumes of gasoline, and a higher number of refueled cars observed in the State of Mexico and Mexico City compared to those in Guadalajara, it is inferred that the vapor recovery system led to a reduction of >50% in gasoline vapor emissions. This reduction rate aligns with findings from Vietnam, where the implementation of vapor recovery systems resulted in a 45% decrease in VOC emissions during vehicle refueling [38].

**Figure 5:**
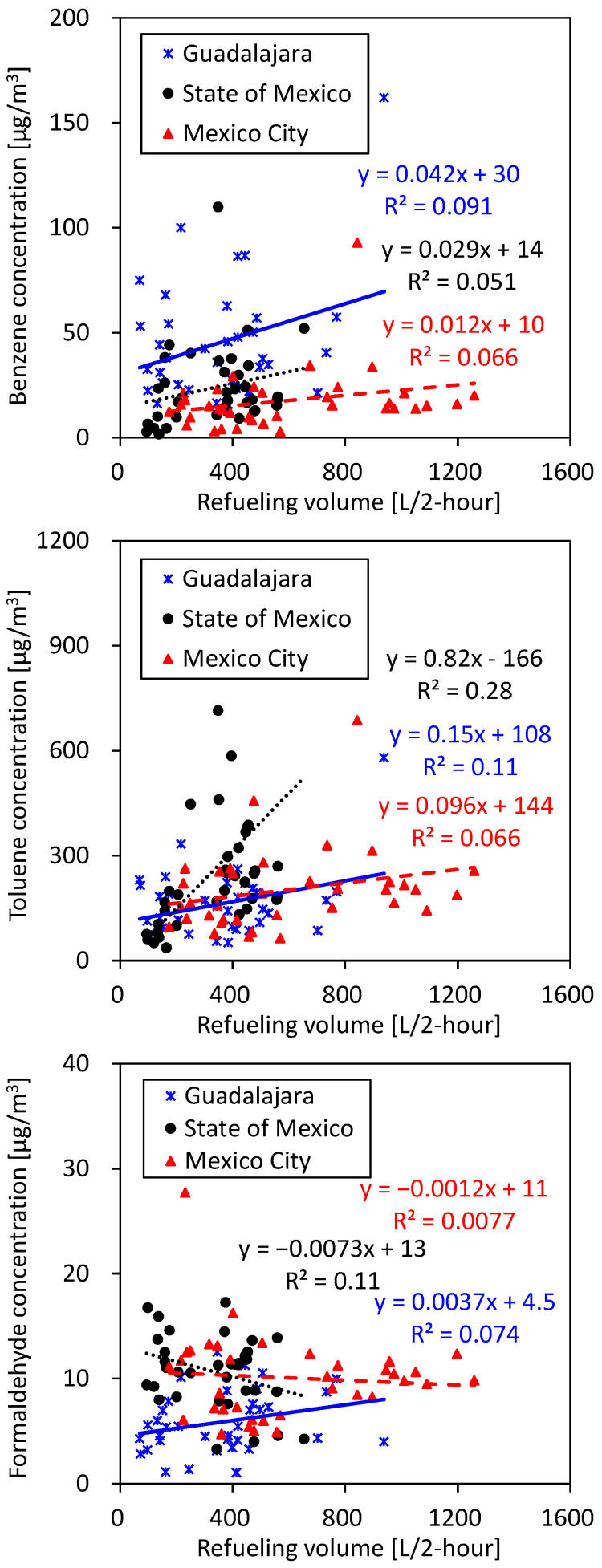
Correlations between exposure concentrations and refueling volume.

Employee exposure in gasoline stations can be either from gasoline vapor or from exhaust gas sources, but they cannot be measured in isolation. Although exposures related to employee health risks are affected by both, the vapor recovery system can reduce exposure only to the components of gasoline vapor. Therefore, PM_2.5_, CO, and formaldehyde in exhaust gases would not be reduced by the vapor recovery system. In this study, many short-time exposures for 2–5 min to CO, which is an incomplete combustion product, in exhaust fumes were observed. This suggested that employees were also exposed to exhaust fumes from vehicles coming to refuel. As VOCs such as BTEX are contained not only in the gasoline vapor but also in the exhaust fumes, some of the exposure to VOCs seen even at gasoline stations in Mexico City and the State of Mexico, where vapor recovery equipment is installed, may be due to exposure to exhaust fumes. This indicates that, in order to reduce exposure to these pollutants, it is also necessary to develop the technology to reduce pollutants in exhaust gas, in addition to installing vapor recovery equipment at gasoline stations. Weak correlations were observed between exposure concentrations and refueling volume (Figure 5), with no discernible correlation observed between exposure concentrations and the number of refueled cars (Figure S8). This weak correlation could potentially be attributed to variations in the duration spent near the open fuel hatch, which may vary depending on individual employee habits and timing. For instance, employees occasionally step away from the hatch during refueling to attend to tasks such as cleaning car windows, thereby introducing variability in exposure durations.

**Figure 6:**
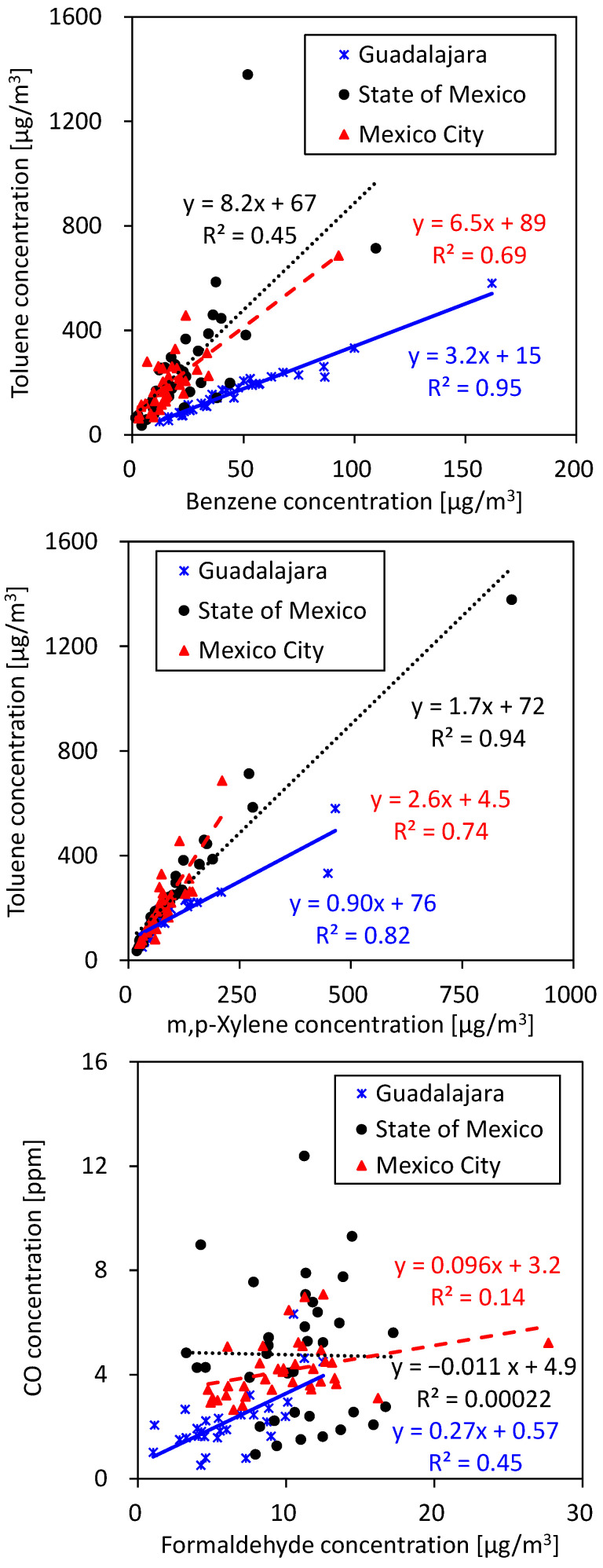
Correlations between exposure concentrations of benzene and toluene, toluene and m,p-xylene, and formaldehyde and CO.

The correlations between exposure concentrations of benzene and toluene, toluene and m,p-xylene, and formaldehyde and CO are shown in Figure 6. The toluene/benzene ratio of exposure levels at gasoline stations in Guadalajara (3.7 ± 0.44 [*w*/*w*]) was similar to that reported for Mexico City in 1999–2001 (3.6–3.9 [43,44]). However, the toluene/benzene ratios of exposure levels at gasoline stations in the State of Mexico and Mexico City (13 ± 7.8 and 14 ± 7.7 [*w*/*w*], respectively) were considerably higher. In particular, high toluene/benzene ratios were observed at gasoline stations SM3, CDMX1, and CDMX2, (57 ± 14, 18 ± 7.7, and 17 ± 4.9, respectively).

Near these gasoline stations, atmospheric toluene concentrations (43 ± 11, 13 ± 7.3, and 9.5 ± 1.8 μg/m^3^, respectively) were much higher compared to the background concentrations (4.6 ± 2.1 μg/m^3^), indicating potential toluene emission sources such as nearby factories. In addition, the toluene/benzene ratio in vapor from regular gasoline is 3.7, whereas for premium gasoline it is 12 [45]. In Guadalajara, the proportion of premium gasoline refueled at stations was 6.3% ± 1.4%. Although the proportions of premium gasoline usage were not documented for gasoline stations in Mexico and Mexico City, it is plausible that the consumption of premium gasoline was higher in those regions.

The volatility of VOCs from fuel increases with higher temperatures. On the other hand, VOCs, CO, and particle matter in exhaust gas produced by combustion are thought to be more likely to cause incomplete combustion at lower temperatures, and the concentration in exhaust gas is higher [46]. In the results of this study, for exposure during refueling, the contribution of volatilization is thought to be large for benzene, toluene, ethylbenzene, and xylenes, and the effect of exhaust gas is thought to be small, so the exposure concentration may be higher in summer than in this survey conducted in spring. On the other hand, CO and PM_2.5_ may be higher in winter than in the current survey. The spread of VOCs from sources such as gas stations to the surrounding area is thought to be greatly affected by wind direction and speed [14,47]. On days with low wind speeds, the pollutants tend to linger in the gasoline station, and the exposure of employees refueling is high, while on days with high wind speeds, the pollutants are diluted, and the exposure of employees is low.

The mean and maximum excess cancer risks were 0.46–4.2 × 10^−5^ and 0.24–1.5 × 10^−4^, respectively. These values are below the threshold of 10^−4^, which typically prompts urgent countermeasures for small population sizes in the US [48]. The risk to employees at gasoline stations in Mexico, including Guadalajara, is not sufficiently high calculated using mean concentrations in the present study. However, the risk calculated using maximum concentrations slightly exceeds 10^−4^. This suggests that it would be better for petrol stations in Guadalajara to take some kind of action, including the installation of a vapor recovery device. As the exposure concentration may vary greatly depending on the season, it will be necessary to conduct surveys in other seasons in the future to obtain the annual mean exposure concentrations.

Implementing countermeasures, such as gas recovery systems, could effectively halve exposure to benzene. Additionally, countermeasures such as installing tight covers for the refueling inlet of car gasoline tanks may prove beneficial in reducing exposure. The excess cancer risk from benzene exposure at gasoline stations was slightly higher than the findings from Japanese gasoline stations lacking vapor recovery systems (2.2 × 10^−5^) [18]. However, in Sri Lanka [20] and Thailand [49], the median benzene exposure among gasoline station employees was apparently higher, potentially resulting in risks of an order of magnitude higher than those observed in gasoline stations in Japan and Mexico. Regarding non-carcinogenic compounds, even at maximum average exposure concentrations, the HQ values for toluene, ethylbenzene, xylenes, and CO were much <1 in Mexico, indicating a low non-carcinogenic risk, similar to that observed in Japan [18]. Conversely, in Sri Lanka [20] and Thailand [49], the HQ values for these non-carcinogenic compounds were >1. This suggests that, in addition to vapor recovery equipment, additional measures such as gasoline reforming may be necessary in certain countries or regions.

In recent years, there has been a move towards more detailed risk assessments, such as the California EPA’s risk assessment, which breaks down weight and respiratory volume into more detailed categories [50]. However, in the present study, no elaborated assessment of body weight or respiratory volume was conducted, as the study was conducted on adults, i.e., employees, and not on children. In the future, assessments can be conducted based on individual characteristics by industry sector.

This study had some limitations, such as the dataset not being large enough, the weather conditions data being unknown, and the fact that the study was not conducted in different seasons. It would be desirable to conduct further studies in the future to enable more detailed evaluations.

## 5. Conclusions

Herein, we compared employee exposure concentrations of BTEX, formaldehyde, CO, and PM_2.5_ at six gasoline stations with gasoline vapor recovery systems to three gasoline stations without such systems. For formaldehyde and PM_2.5_, employee exposure concentrations did not differ from background levels. The average excess cancer risk attributed to benzene exposure among gasoline station employees was mostly between 10^−5^ and 10^−4^, indicating a risk level that, while less demanding of immediate countermeasures in the work environment, exceeds that typically deemed acceptable in the general environment, suggesting the potential necessity for future intervention measures. Conversely, for non-carcinogenic risks, the HQ values remained well below 1, indicating no significant risk concerns. Furthermore, BTEX exposure concentrations at gasoline stations equipped with vapor recovery units were notably lower than those at gasoline stations without such systems, affirming a reduction in exposure concentrations by approximately half.

## Figures and Tables

**Table 1:** Estimated excess cancer risks associated with benzene exposure at gasoline stations in Guadalajara, State of Mexico, and Mexico City.

	Benzene Exposure [μg/m^3^]		Estimated Excess Cancer Risk [Unitless]	
	Mean	Max	Mean	Max
Guadalajara	45	1.6 × 10^2^	1.2–4.2 × 10^−5^	0.42–1.5 × 10^−4^
State of Mexico	24	1.1 × 10^2^	0.63–2.2 × 10^−5^	0.29–1.0 × 10^−4^
Mexico City	18	93	0.46–1.6 × 10^−5^	2.4–8.6 × 10^−5^

## Data Availability

The datasets used or analyzed in this study are available from the corresponding author upon reasonable request.

## Supplementary Materials

The following supporting information can be downloaded at: https://www.mdpi.com/article/10.3390/ijerph22010010/s1, Figure S1: Refueling volume of gasoline for 2 h per employee in (a) Guadalajara, (b) State of Mexico, and (c) Mexico City; Figure S2: Number of refueled cars in 2 h per employee in (a) Guadalajara, (b) State of Mexico, and (c) Mexico City; Figure S3: Employee exposure and background concentrations of VOCs at gas stations in Guadalajara. (a) Benzene, (b) toluene, (c) ethylbenzene, (d) m,p-xylene, (e) o-xylene, and (f) formaldehyde; Figure S4: Personal exposure and background concentrations of VOCs at gas stations in the State of Mexico. (a) Benzene, (b) toluene, (c) ethylbenzene, (d) m,p-xylene, (e) o-xylene, and (f) formaldehyde; Figure S5: Personal exposure and background concentrations of VOCs at gas stations in Mexico City. (a) Benzene, (b) toluene, (c) ethylbenzene, (d) m,p-xylene, (e) o-xylene, and (f) formaldehyde; Figure S6: Personal exposure concentrations of CO at gas stations. in (a) Guadalajara, (b) State of Mexico, and (c) Mexico City; Figure S7: Example of real-time monitoring results of personal exposure and background concentrations of CO at gas stations in Guadalajara (19 May, 2012); Figure S8: Correlation between the exposure concentrations and refueling car number.
