# Supplementary material for: Exposure/Risk Assessment of Employees in Gasoline Refueling Stations with and Without the Efficacy of Vapor Recovery Systems in Mexico
Source: Int J Environ Res Public Health. 2024 Dec 25;22(1):10. doi: 10.3390/ijerph22010010 (PMC11765004; doi:10.3390/ijerph22010010)
Supplement: Supplementary file 1 [file ijerph-22-00010-s001.zip › ijerph-3342776-supplementary.pdf]

Supplementary Materials

For

Exposure/Risk Assessment of Employees in Gasoline Refueling Stations with and Without the Efficacy of Vapor Recovery Systems in Mexico

Naohide Shinohara [a,*], Jose Juan Felipe Ángeles García [b], Miguel Magaña Reyes [b], Becki Gatica Jiménez [b], Roberto Basaldud Cruz [b], Beatriz Cardenas Gonzalez [c], Shinji Wakamatsu [d,e]

[a] Research Institute of Science for Safety and Sustainability (RISS), National Institute of Advanced Industrial Science and Technology (AIST), *16-1 Onogawa, Tsukuba, Ibaraki 305-8569, Japan*

[b] National Institute of Ecology and climate change (INECC), The Secretariat of Environment and Natural Resources (SEMARNAT), Universidad Autónoma Metropolitana - Iztapalapa*, Av. San Rafael Atlixco No. 186, Col. Vicentina, CP 09340, Del. Iztapalapa, CDMX, Mexico*

[c] World Resources Institute Mexico, Belisario Dominguez no. 8, Coyoacan, 04000, CDMX, Mexico

[d] Institute of Integrated Atmospheric Environment, *1-2-8 Koraku, Bunkyo, Tokyo 112-0004, Japan*

[e] Faculty of Agriculture, Ehime University, *10-13 Dogo-himata, Matsuyama, Ehime 790-8577, Japan*

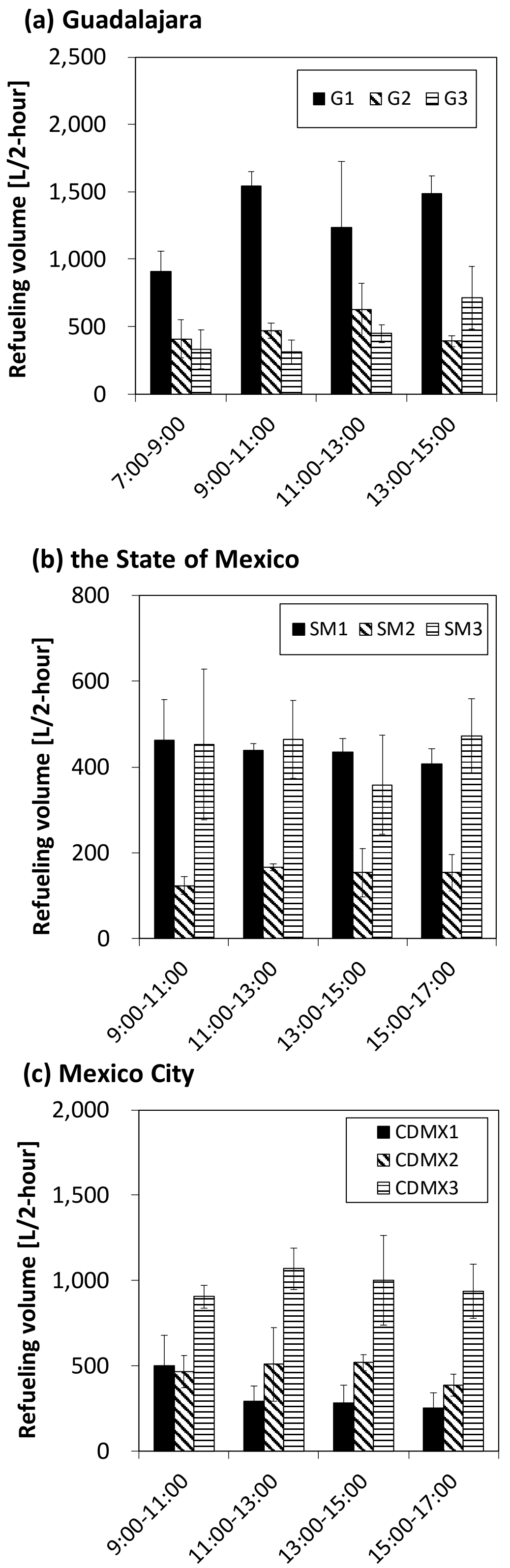

Figure S1 Refueling volume of gasoline for 2 h per employee in (a) Guadalajara, (b) State of Mexico, and (c) Mexico City.

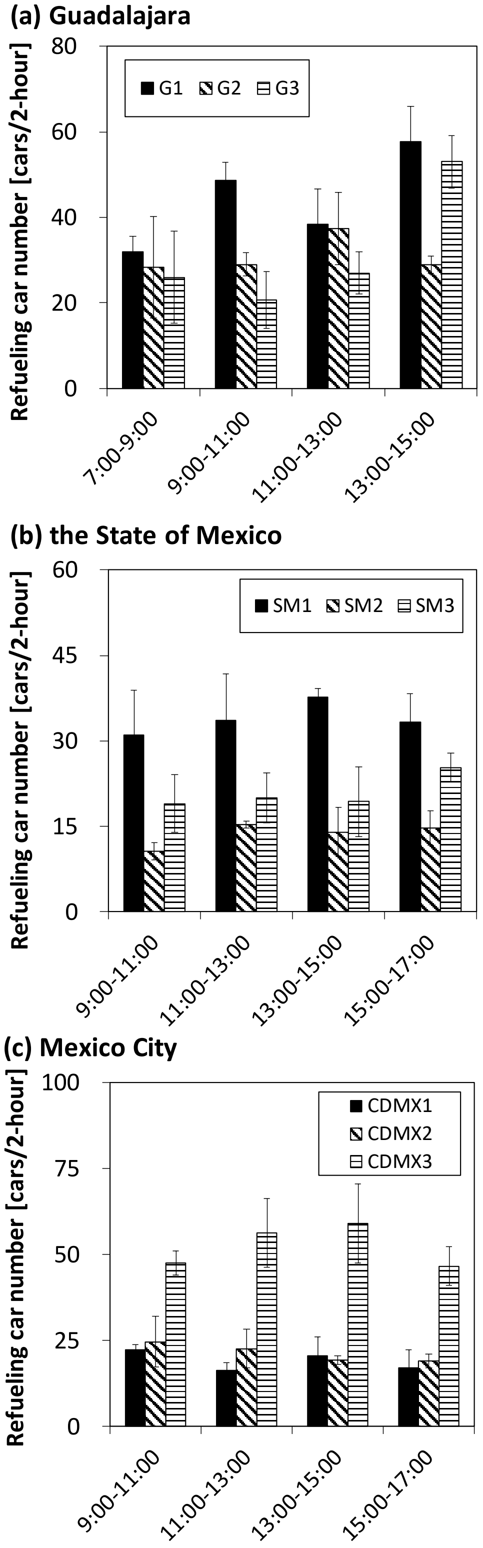

Figure S2 Number of refueled cars in 2 h per employee in (a) Guadalajara, (b) State of Mexico, and (c) Mexico City.

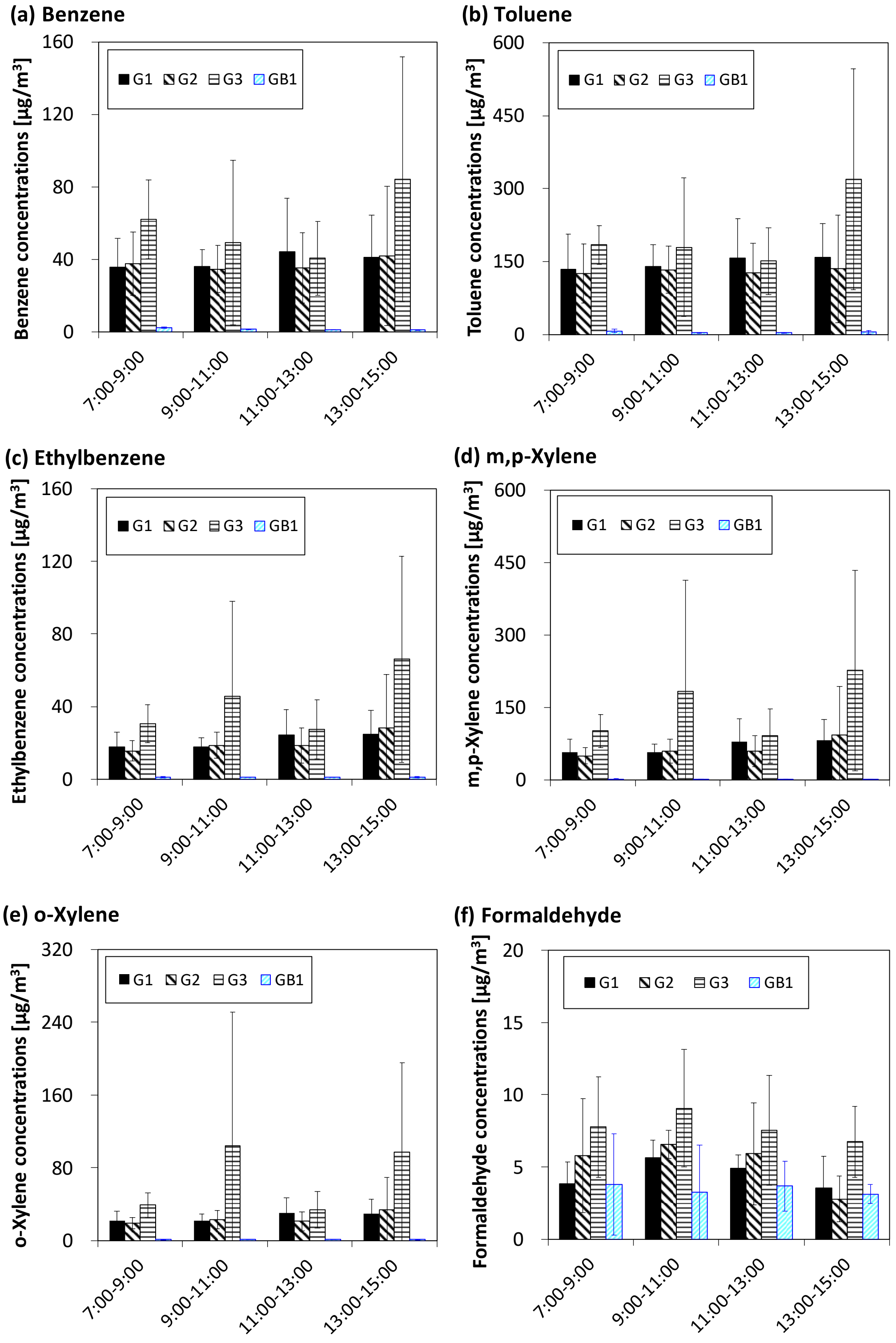

Figure S3 Employee exposure and background concentrations of VOCs at gas stations in Guadalajara. (a) Benzene, (b) toluene, (c) ethylbenzene, (d) m,p-xylene, (e) o-xylene, and (f) formaldehyde.

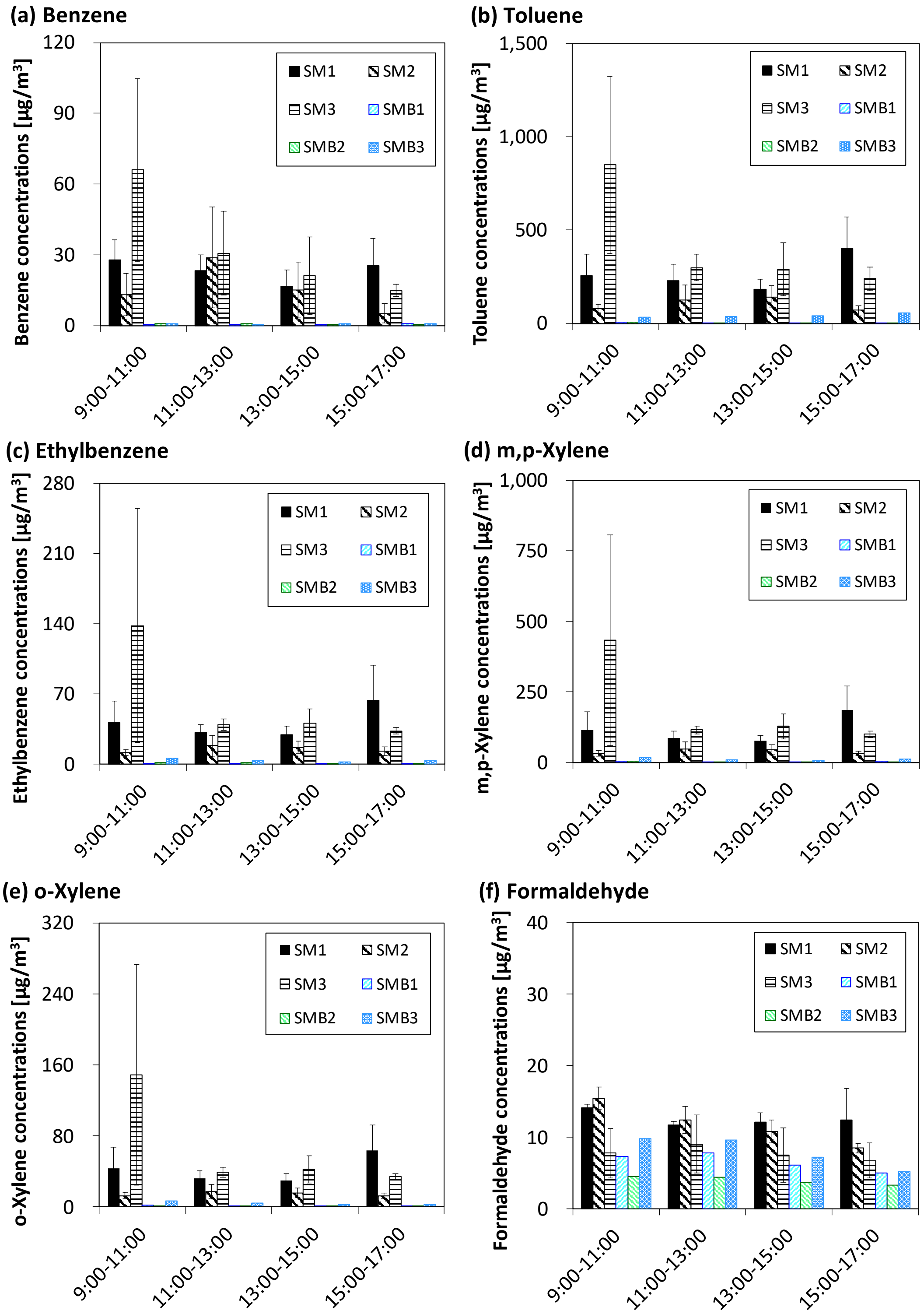

Figure S4 Personal exposure and background concentrations of VOCs at gas stations in the State of Mexico. (a) Benzene, (b) toluene, (c) ethylbenzene, (d) m,p-xylene, (e) o-xylene, and (f) formaldehyde.

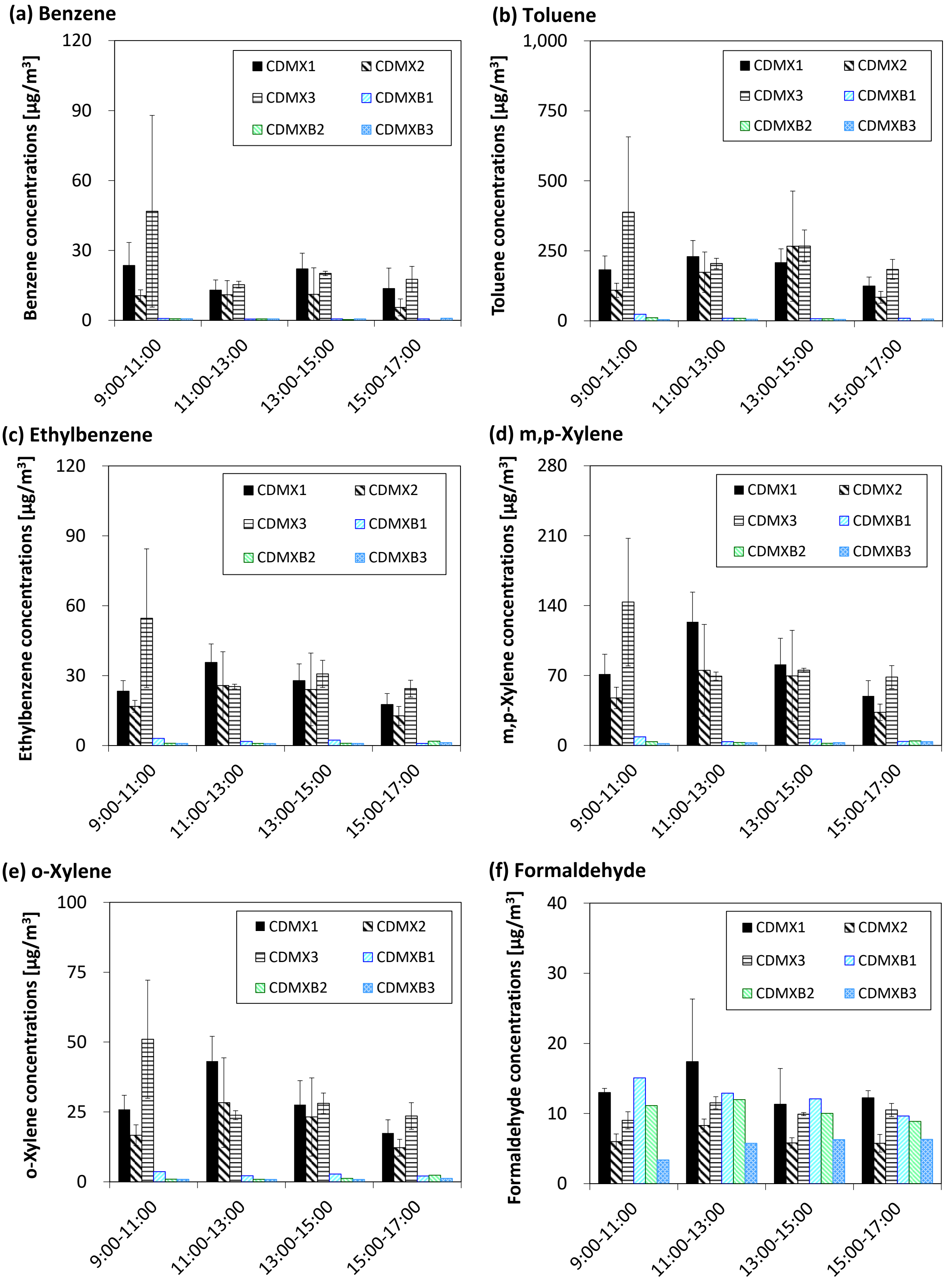

Figure S5 Personal exposure and background concentrations of VOCs at gas stations in Mexico City. (a) Benzene, (b) toluene, (c) ethylbenzene, (d) m,p-xylene, (e) o-xylene, and (f) formaldehyde.

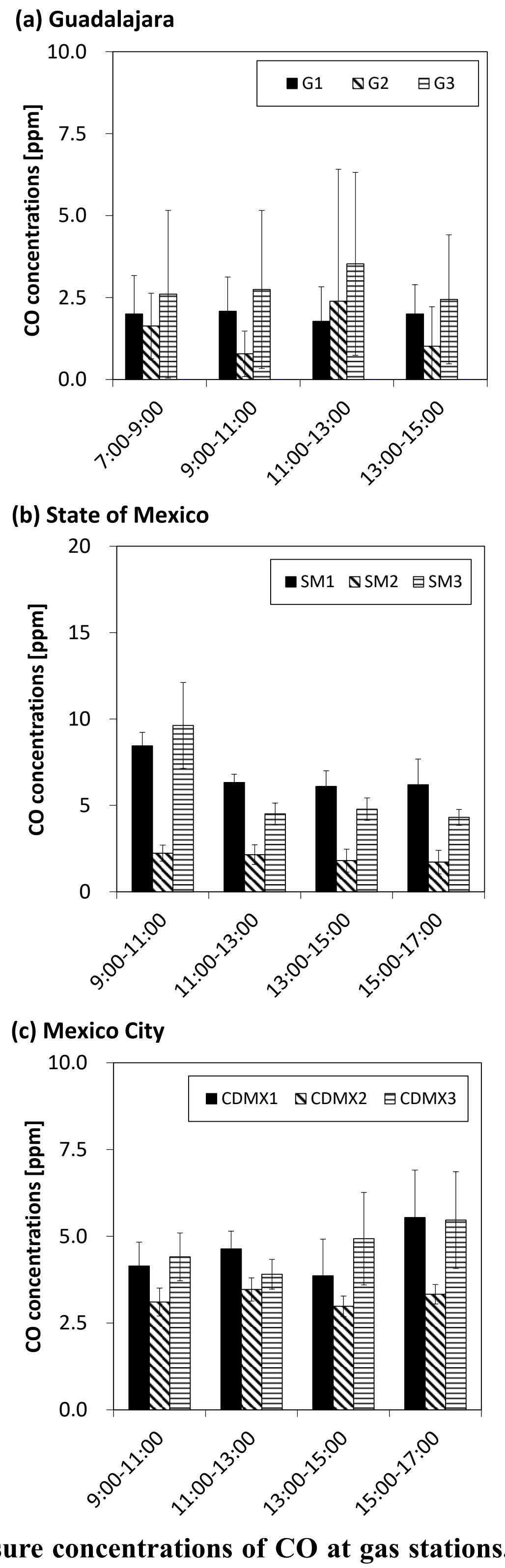

Figure S6 Personal exposure concentrations of CO at gas stations. in (a) Guadalajara, (b) State of Mexico, and (c) Mexico City.

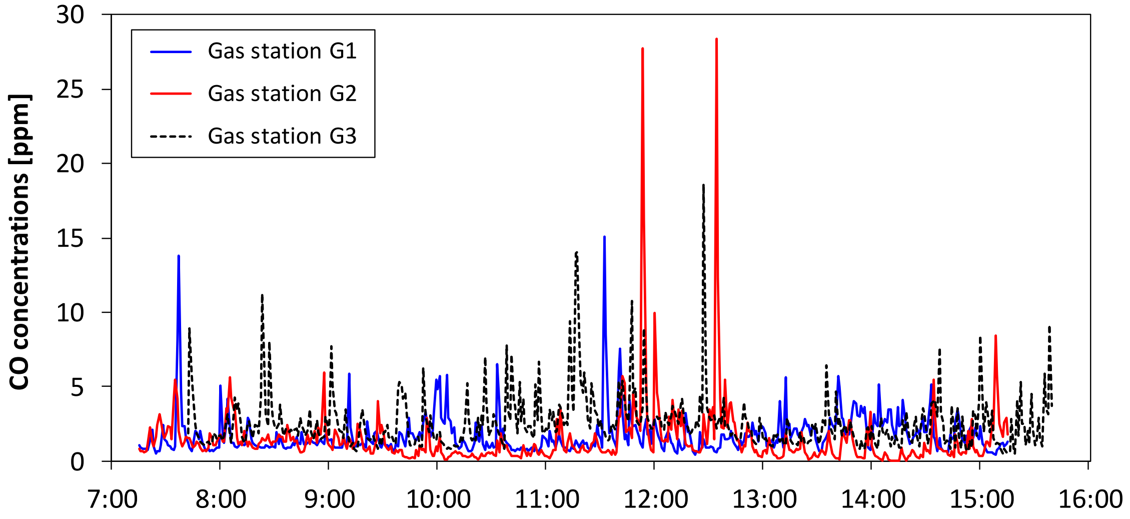

Figure S7 Example of real-time monitoring results of personal exposure and background concentrations of CO at gas stations in Guadalajara (19 May, 2012).

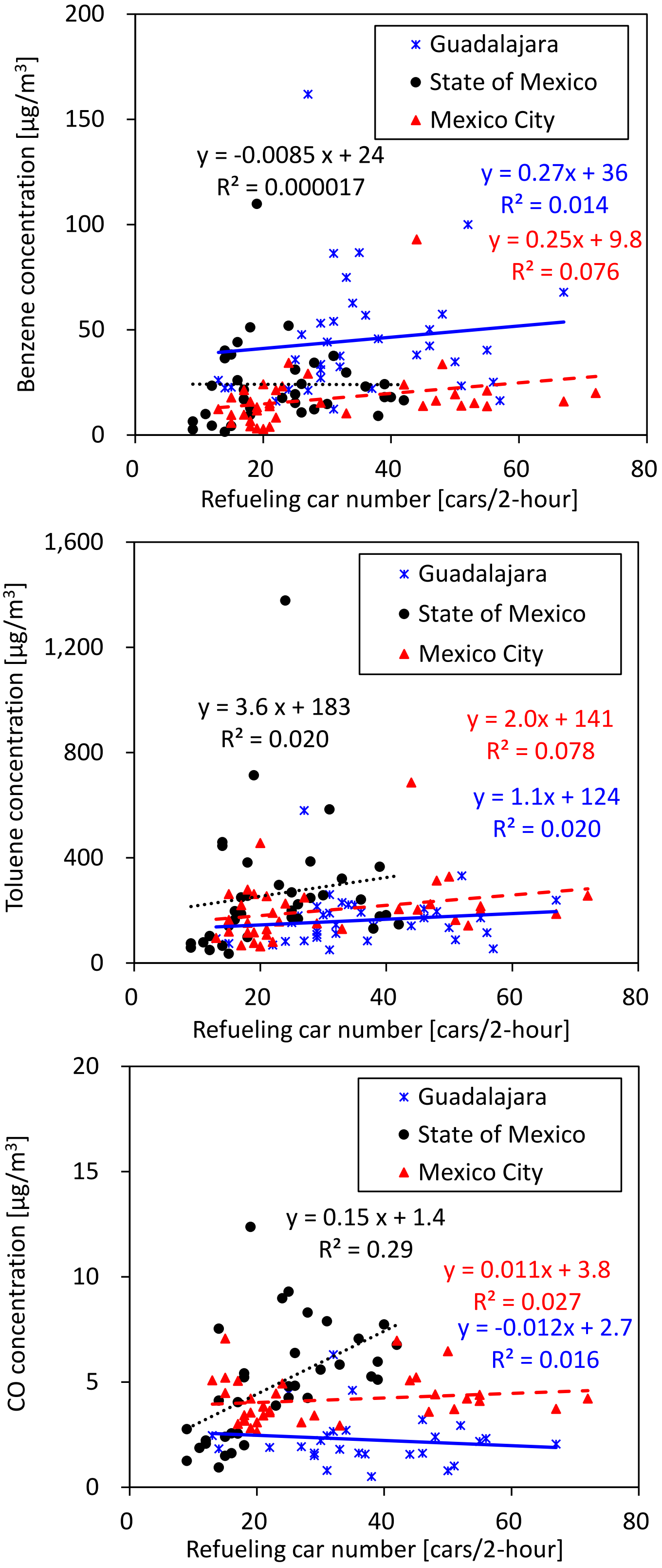

Figure S8 Correlation between the exposure concentrations and refueling car number.
